# Effects of selenium-mediated RUNX2 overexpression and its transcriptome alterations on Chondrocyte injury in Kashin Beck disease

**DOI:** 10.3389/fcell.2025.1687954

**Published:** 2025-12-05

**Authors:** Di Zhang, Qiang Li, Xiaoli Yang, Yongmin Xiong, Shiquan Sun

**Affiliations:** 1 National and Local Joint Engineering Research Center of Biodiagnosis and Biotherapy, The Second Affiliated Hospital of Xi’an Jiaotong University, Xi’an, Shaanxi, China; 2 Institute of Endemic Diseases and Key Laboratory of Trace Elements and Endemic Diseases, School of Public Health, Xi′an Jiaotong University Health Science Center, Xi’an, Shaanxi, China; 3 Shaanxi Provincial Key Laboratory of Infection and Immune Diseases, Shaanxi Provincial People’s Hospital, Xi’an, Shaanxi, China

**Keywords:** Kashin-Beck disease, selenium deficiency, RUNX2, Chondrocyte injury, RNA transcription profiles

## Abstract

**Objective:**

Kashin-Beck disease (KBD) is a nutrition-related osteoarthropathy characterized by excessive apoptosis and matrix destruction. Micronutrient selenium (Se) deficiency is recognized as a major environmental risk factor. This study aimed to investigate the role of RUNX2 in cartilage injury associated with KBD.

**Methods:**

RUNX2 expression in articular cartilage from KBD patients was assessed by immunohistochemistry. *RUNX2* mRNA expression and promoter methylation were analyzed using qRT-PCR and qMSP. A chondrocyte injury model was established under Se-deficient conditions, while a *RUNX2* overexpression model was generated by lentiviral transfection and further analyzed by RNA sequencing.

**Results:**

The proportion of RUNX2-positive cells in KBD cartilage was significantly higher than in controls. *RUNX2* mRNA levels were elevated, whereas methylation levels were reduced in KBD samples. Inhibition of DNA methylation confirmed that decreased methylation of *RUNX2* promoted its transcription. In Se-deficient chondrocytes, decreased *RUNX2* methylation, increased RUNX2 expression, and higher rates of apoptosis and necrosis were observed, all of which were reversed by Se supplementation. Moreover, RUNX2 overexpression further increased chondrocyte apoptosis and necrosis. Transcriptomic analysis revealed 263 upregulated and 216 downregulated genes, predominantly enriched in the TNF and MAPK signaling pathways.

**Conclusion:**

Micronutrient Se deficiency may contribute to the pathogenesis of KBD by modulating RUNX2 expression and inducing excessive chondrocyte apoptosis, while Se supplementation exerts a protective effect. RUNX2 plays a critical role in KBD progression and may represent a potential therapeutic target.

## Graphical Abstract

**Figure:**
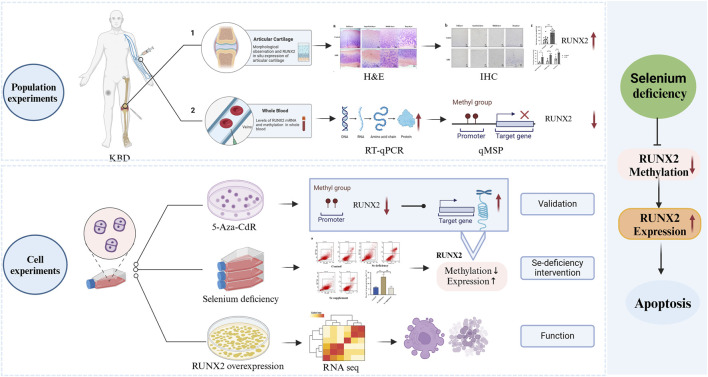

## Introduction

Kashin-Beck disease (KBD) is an endemic osteoarthropathy characterized by chronic degeneration, necrosis, and secondary alterations in hyaline cartilage (Guo et al., 2014; Zhang et al., 2018). Clinically, patients typically present with joint pain, impaired mobility, and joint deformities (Xiong, 2001). In China, approximately 164,321 individuals have been diagnosed with grade I or higher KBD, and more than 100 million people remain at risk, imposing a substantial socio-economic burden (Commission NHAF, 2020). Despite extensive research, the precise pathogenesis of KBD remains unclear, underscoring the need to investigate its etiology further and identify potential therapeutic targets.

Selenium (Se) is an essential micronutrient with diverse biological functions and is indispensable for human health. Globally, an estimated 0.5 to 1 billion people are affected by Se deficiency due to insufficient dietary intake (Radomska et al., 2021). Epidemiological studies have shown that KBD is most prevalent in the Siberian region of Russia, northern North Korea, and a narrow belt extending from northeast to southwest China, where its geographic distribution overlaps with areas deficient in selenium. Moreover, crops grown in Se-deficient soils have been identified as a significant environmental risk factor for KBD (Han et al., 2025; Wu et al., 2024). Zhang et al. further reported that Se levels in both flour and plasma were significantly lower in KBD patients than in controls, indicating Se deficiency in both the external environment and the internal milieu of affected individuals (Zhang et al., 2023). Nevertheless, the specific molecular mechanisms by which Se contributes to KBD pathogenesis remain poorly understood and require further investigation.

Gene-environment interactions play a crucial role in the pathogenesis of KBD (Yang et al., 2022a). Research on cartilage-related genes has become a major focus in the field of osteoarthropathy. Runt-related transcription factor 2 (RUNX2) is a key regulator of osteoblast and chondrocyte differentiation and is indispensable for chondrocyte maturation (Hallett et al., 2025). Previous studies have established a strong association between RUNX2 and the progression of osteoarthritis (OA). For example, Runx2^+/−^ mice were resistant to OA progression, whereas chondrocyte-specific deletion of RUNX2 slowed OA development (Liao et al., 2017). Conversely, chondrocyte-specific RUNX2 overexpression accelerates post-traumatic OA progression in adult mice (Catheline et al., 2019). In KBD, cartilage injury is characterized by excessive chondrocyte apoptosis and degradation of the extracellular matrix. However, the role of RUNX2 in KBD pathogenesis remains poorly understood.

Epigenetic variation serves as a critical molecular link between environmental factors and disease (Cavalli and Heard, 2019). Among epigenetic mechanisms, DNA methylation plays a crucial role in gene regulation and cell differentiation (Miranda-Duarte, 2018). Aberrant DNA methylation has been implicated in the pathogenesis of numerous disorders, including cardiovascular disease, diabetes, and OA (Qin et al., 2019; Rönn and Ling, 2015; Ratneswaran and Kapoor, 2021). Notably, RUNX2 expression has been reported to be negatively correlated with CpG island methylation in patients with HOX11^(+)^ acute T-lymphoblastic leukemia (Jia and Salvatore, 2013). Increasing evidence suggests that gene–environment interactions are central to the development of KBD. For example, low Se levels in crops have been shown to alter methylation patterns of environmentally susceptible genes in humans (Jiang et al., 2021). However, the potential interplay between Se deficiency, region-specific RUNX2 methylation, and subsequent RUNX2 expression in KBD remains poorly understood. Thus, whether Se deficiency contributes to cartilage injury in KBD by modulating RUNX2 warrants further investigation.

In this study, we evaluated RUNX2 expression in articular cartilage and peripheral blood samples from KBD patients and analyzed the levels of RUNX2 promoter methylation. To clarify the relationship between RUNX2 expression and methylation, a demethylation model was established using 5-aza-2′-deoxycytidine (5-Aza-CdR). In addition, a chondrocyte injury model induced by Se deficiency was employed to determine whether environmental risk factors contribute to KBD pathogenesis through RUNX2. Furthermore, RUNX2 was overexpressed in chondrocytes and subsequently analyzed by RNA sequencing (RNA-seq) to investigate its role in chondrocyte injury and associated signaling pathways. Collectively, this study aimed to provide new insights into the etiology and pathogenesis of KBD and to identify RUNX2 as a potential therapeutic target for KBD.

## Methods

### Study population

According to the criteria of the National Health Commission of the People’s Republic of China (WS/T-207-2010), X-ray examination and clinical diagnosis, a total of 103 KBD patients and 109 controls were included, and whole blood was collected. Five KBD knee cartilage samples came from KBD patients undergoing joint replacement surgery, and five normal knee cartilage samples came from post-traumatic amputation patients in the hospital. Controls and patients were comparable with respect to age and sex, and all subjects are Han Chinese without other joint diseases. All subjects signed informed consent before the experiment.

### Hematoxylin-eosin staining

The cartilage samples were embedded in paraffin and sectioned into 5-μm-thick slices. Paraffin sections of cartilage were dewaxed in xylene before dehydration with gradient ethanol. The sections were stained with hematoxylin-eosin (H&E) to assess the morphology changes of KBD cartilage using an optical microscope (Olympus, Tokyo, Japan).

### Immunohistochemical staining

The cartilage paraffin sections were treated with 0.3% H_2_O_2_ for 20 min, then incubated with trypsin for 20 min and placed in 1X sodium citrate for 4 h. The sections were blocked using normal goat serum (ZSGB-BIO, Beijing, China) for 15 min, followed by incubation with RUNX2 primary antibody (Immunoway, Texas, United States) at a 1:300 dilution overnight at 4 °C. Sections were incubated with Biotin-labeled goat anti-rabbit IgG polymer and then with horseradish peroxidase for 15 min. Finally, incubation with 3,3′-diaminobenzidine reagent (ZSGB-BIO, Beijing, China). The images were obtained using a Leica slide scanning microscope (Leica SCN400, Weztlar, Germany). Each cartilage slice was divided into three regions: superficial, middle and deep. The criteria we use to distinguish the superficial, middle and deep layers are cell morphology, density, arrangement and aggregation. Five non-repetitive regions of shallow, medium and deep regions were obtained from each sample; 75 images were collected in each group, and the three layers were analyzed. The cells in the cytoplasm were counted by ImageJ. The positive rate was calculated as follows:

$$\begin{array}{ll} & \text{The percentage of positive cells}\\ & \;=\frac{\text{positive}\text{staining}\text{cells}}{\text{positive}\text{staining}\text{cells}+\text{negative}\text{staining}\text{cells}}\times 100\% \end{array}$$

### Establishment of a chondrocyte injury model by selenium deficiency

The human C28/I2 chondrocyte line was provided by Dr Mary B Goldring from Cornell University, United States (Claassen et al., 2011). The injury model was established using Se deficiency. Chondrocytes were conditioned with DMEM Ham’s F-12 medium supplemented with 1% fetal calf serum (SiJiQing Bio-Technique Co., Ltd, Zhejiang, China) and 1% penicillin-streptomycin solution under a 5% CO_2_ atmosphere for 5 days, resulting in Se depletion (Yan et al., 2013). Chondrocytes were treated with different concentrations (10, 30, 50, 70, 90, 110, 130 nM) of Na_2_SeO_3_, then 90 nM Na_2_SeO_3_ was selected as the optimal concentration.

### The overexpression of *RUNX2* in chondrocytes

Chondrocytes were seeded into 6-well plates and cultured overnight in an incubator. The human-RUNX2 overexpression lentivirus (HBLV-h-RUNX2-3xflag-ZsGreen-PURO) and negative control lentivirus (HBLV-ZsGreen-PURO) were added to transfect cells, which were designed and synthesized by HanBio (Hanbio, Shanghai, China). The optimal multiple of infection (MOI) was selected as 40, the solution of polybrene was 6 μg/mL, and the infection was maintained for 72 h in the lentivirus infection experiment. The stable transfection cells were screened using 1.5 μg/mL puromycin for 5 days, and the overexpression efficiency was detected by quantitative real-time PCR (qRT-PCR).

### Identification and enrichment analysis of DEGs

Total RNA was extracted using the Trizol reagent kit (Invitrogen, CA, United States) according to the manufacturer’s protocol. The RNA quality was assessed using an Agilent 2,100 Bioanalyzer (Agilent Technologies, CA, United States) and verified by RNase-free agarose gel electrophoresis. The RNA was reverse-transcribed into cDNA using the NEBNext Ultra RNA Library Prep Kit (NEB #7530, New England Biolabs, MA, United States). The cDNA libraries were sequenced on the Illumina sequencing platform by Genedenovo Biotechnology Co., Ltd (Guangzhou, China).

In total, six RNA samples (NC and RUNX2 overexpression samples) were analyzed. Each sample generated approximately 6 Gb of clean data on average. The overall sequencing quality was evaluated using FastQC, and all samples passed the standard thresholds for base quality, GC content, and read length distribution, ensuring data reliability for subsequent analyses. Low-quality reads and inferior-quality bases from the raw data were removed and filtered using fastp (version 0.18.0) (Chen et al., 2018). Then, the Short Reads Alignment tool Bowtie2 (version 2.2.8) was used for mapping reads to the ribosomal RNA (rRNA) database (Langmead and Salzberg, 2012). The clean data were then aligned to the human reference genome (GRCh38) by HISAT2 (Kim et al., 2015), and the mapped reads of each sample were assembled by using StringTie v1.3.1 in a reference-based approach (Pertea et al., 2015; Pertea et al., 2016). A FPKM (fragment per kilobase of transcript per million mapped reads) value was calculated to quantify its expression abundance and variations using RSEM software (Li and Dewey, 2011). Finally, the DESeq2 package was applied to analyze differentially expressed genes (DEGs). Enrichment analyses of DEGs were implemented by Gene Ontology (GO) (Ashburner et al., 2000), Kyoto Encyclopedia of Genes and Genomes (KEGG) pathway (Ogata et al., 1999), and Disease Ontology (DO) (Schriml et al., 2012), and *P*-value < 0.05 was regarded as the threshold.

### Quantitative methylation-specific PCR

DNA was extracted using a DNA Kit (Tiangen, Beijing, China). Methylation-specific bisulfite conversion was performed by EZ-DNA methylation Kit (ZYMO, CA, United States). The reactions of quantitative methylation-specific PCR (qMSP-PCR) were performed in a 10.0 μL mixture, including 1.00 μL DNA, 3.2 μL ddH_2_O, 0.40 μL each primer (10 μM), and 5.00 μL TB Green™ (Takara, Kusatsu, Japan). The primers’ sequences are shown in Supplementary Table S1, which were synthesized by Huada (BGI, Shenzhen, China).

### Flow cytometry

Apoptotic chondrocytes were quantified by flow cytometry using an Annexin V-fluorescein isothiocyanate (FITC)/propidium iodide (PI)-Apoptosis detection kit (BD, NJ, United States). The chondrocytes treated with the sample were collected and resuspended in 1× binding buffer. Then, 5.0 µL of Annexin V-FITC and 5.0 µL of PI were added, and the mixture was incubated for 15 min. Finally, 200 μL of 1× binding buffer was added to the stained cells, and the cells were analyzed using Cell Quest software version 1.0 (BD, NJ, United States).

### Quantitative real-time PCR

According to the manufacturer’s instructions, total RNA was isolated using the Trizol reagent (Invitrogen, CA, United States). A total of 500 ng RNA was reverse transcribed in a thermocycler using the Revert AidTM First Strand complementary DNA Synthesis Kit (Thermo Scientific, MA, United States). The reactions of qRT-PCR were performed in a 13.0 μL mixture, including 1.00 μL DNA (50 ng/μL), 4.46 μL ddH_2_O, 0.52 μL each primer (10 μM), and 6.50 μL TB Green™. The primers’ sequences are shown in Supplementary Table S2.

### Statistical analysis

The measurement data were expressed as ±SD. The data, consistent with both normal distribution and homogeneity of variance, are statistically analyzed using a two-tailed t-test and variance analysis. The data with a skewed distribution are tested using a nonparametric test. The counting data were expressed as sample frequencies, and the differences between groups were analyzed using the chi-square test or Fisher’s exact probability method. α = 0.05 was used as the test level, and the data were analyzed using SPSS 23.0.

## Results

### Demographic characteristics of the study population

Among the collected samples (103 KBD patients and 109 controls), 20 subjects were randomly selected for demographic data presentation and subsequent analysis. The baseline characteristics of KBD patients and healthy controls are shown in Supplementary Table S3. There were no significant differences between the KBD group and the control group for cartilage and blood samples in terms of age (*P* = 0.793, *P* = 0.907) and gender (*P* = 0.835).

### Morphological observation and RUNX2 *in situ* expression of articular cartilage in KBD patients

**FIGURE 1:**
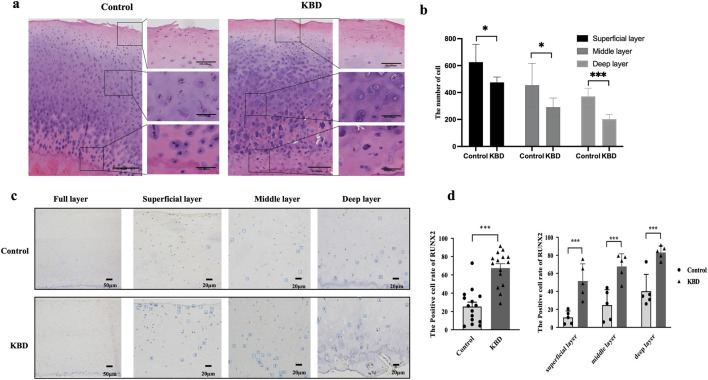
Morphological observation and RUNX2 expression of articular cartilage in KBD patients. **(a)** Representative image of H&E staining in articular cartilage of KBD patients. **(b)** The number of chondrocyte in articular cartilage. **(c)** Representative images of RUNX2 immunohistochemistry staining in articular cartilage of KBD patients. **(d)** The RUNX2 positive percentages in articular cartilage of KBD patients. Data on the graph are shown as mean ± SD; *** Compared with the control group, *P* < 0.0001.

Histological sections of articular cartilage from control and KBD groups were stained with H&E, and morphological changes were examined under an optical microscope (Figure 1a). In the control group, chondrocytes appeared smooth, intact, and orderly arranged. In contrast, KBD cartilage exhibited a significant reduction in chondrocyte numbers across the superficial, intermediate, and deep layers (Figure 1b). Chondrocytes are irregularly distributed, with numerous lacunae and areas lacking normal structure observed in the deep layers.

IHC staining revealed RUNX2-positive areas as brown signals, with positive cells distributed throughout the articular cartilage of KBD patients (Figure 1c). The proportion of RUNX2-positive cells was significantly higher in the KBD group compared with controls (*P* < 0.0001). Stratified analysis showed that the percentages of RUNX2-positive cells in the superficial, middle, and deep layers of KBD cartilage were all significantly higher than those in the corresponding layers of controls (all *P* < 0.001; Figure 1d; Supplementary Table S4). Moreover, within the KBD cartilage, the proportion of RUNX2-positive cells in the deep layer was higher than that in the superficial and middle layers (*P* = 0.0019 and *P* = 0.0243, respectively).

### Expression of RUNX2 and related genes in the blood of KBD patients

**FIGURE 2:**
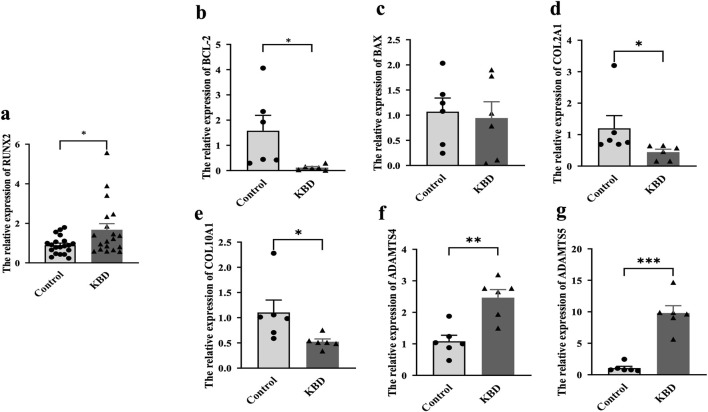
Expression of RUNX2 and related genes in whole blood of KBD patients. **(a)** The mRNA level of RUNX2 in KBD patients. **(b–g)** The mRNA levels of related genes (BCL-2, BAX, COL2A1, COL10A1, ADAMTS4, ADAMTS5) in whole blood of KBD patients. Data on the graph are shown as mean ± SD; * Compared with the control group, *P* < 0.05; ** Compared with the control group, P < 0.001; *** Compared with the control group, *P* < 0.0001.

The results showed that the expression of *RUNX2* in the whole blood of KBD patients was significantly higher than that of the controls (*P* = 0.0193), as shown in Figure 2a, which was consistent with the results of KBD cartilage. Furthermore, we also detected the expression of cartilage injury-related genes in KBD patients (Figures 2b–g). The results showed that the expression of *BCL-2* was lower in KBD patients than in the controls (*P* = 0.0380). In contrast, the expression of *BAX* showed no difference between the two groups (*P* = 0.2114). The results also showed that the mRNA levels of *COL2A1* and *COL10A1* were decreased in the KBD group (*P* = 0.0396, *P* = 0.0486), while *ADAMTS4* and *ADAMTS5* were increased in the KBD group (*P* = 0.0015, *P* < 0.0001).

### Effect of *RUNX2* DNA methylation on gene expression

**FIGURE 3:**
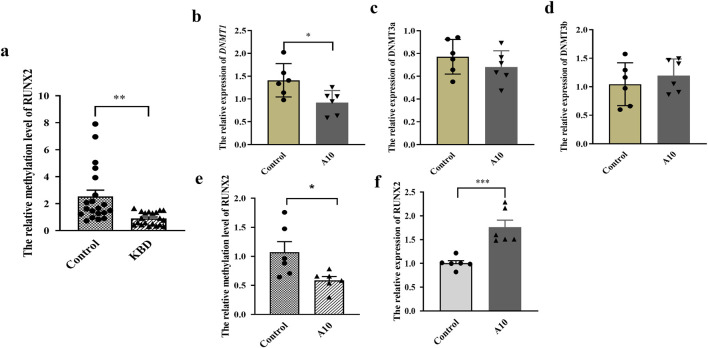
Effect of RUNX2 DNA methylation on gene expression. **(a)** The methylation level of RUNX2 in whole blood of KBD patients. **(b–d)** The mRNA level of DNMTs (DNMT1, DNMT2a, DNMT2b) in chondrocytes treated with 5-Aza-CdR. **(e)** The methylation level of RUNX2 in chondrocytes treated with 5-Aza-CdR. **(f)** The mRNA level of RUNX2 in chondrocytes treated with 5-Aza-CdR. A10: treated with 10 μg/mL of 5-Aza-CdR for 72h; Data on the graph are shown as mean ± SD; * Compared with the control group, *P* < 0.05; *** Compared with the control group, *P* < 0.0001.

The results showed that the methylation level in the promoter region of *RUNX2* in KBD patients was significantly lower than that of controls (*P* = 0.0014), as shown in Figure 3a. To explore the effect of *RUNX2* DNA methylation on gene expression, a methylation inhibition experiment was conducted. Chondrocytes were treated with 10 μg/mL of 5-Aza-CdR for 72 h. The results showed that the mRNA level of *DNMT1* in the 5-Aza-CdR group was decreased than that of the control group (*P* = 0.0243), while there was no difference in the expression of *DNMT3a* and *DNMT3b* (*P* = 0.3119, *P* = 0.4500), shown in Figures 3b–d. The methylation level of *RUNX2* was decreased significantly in the 5-Aza-CdR group (*P* = 0.0318) while the mRNA level of *RUNX2* was increased (*P* = 0.0008), shown in Figures 3e,f.

### Selenium deficiency induces RUNX2 expression in chondrocytes

**FIGURE 4:**
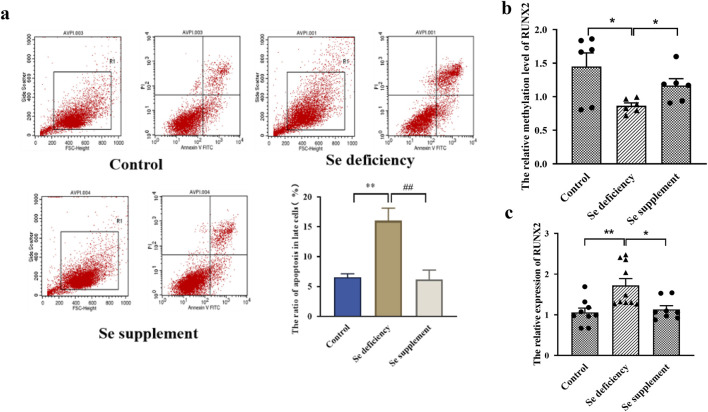
Effects of RUNX2 expression on chondrocyte apoptosis induced by selenium deficiency. **(a)** representative image of Annexin V-FITC/PI staining and the apoptosis and necrosis rates in chondrocytes induced by Se deficiency. **(b)** The methylation level of RUNX2 in chondrocytes induced by Se deficiency. **(c)** The mRNA level of RUNX2 in chondrocytes induced by Se deficiency. Se deficiency: 1% FBS treated for 7days; Se supplement: 1% FBS treated for 5days and then 90 nM Na2SeO3 for 2days; Data on the graph are shown as mean ± SD; * Compared with the control group, P < 0.05; ** Compared with the control group, P < 0.001.

For experiments on Se deficiency, the Annexin V-FITC/PI staining results showed that the late apoptosis rate in the Se-deficient group was higher than that of the control group (*P* = 0.0024). In comparison, the late apoptosis rate of the Se supplement group was lower than that of the Se deficiency group (*P* = 0.0031) in Figure 4a. Furthermore, the mRNA level of *RUNX2* was increased. The methylation level was decreased in the Se deficiency group (*P* = 0.0046, *P* = 0.0174). In contrast, a low expression and high methylation level of *RUNX2* were observed in the Se supplement group (*P* = 0.0110, *P* = 0.0208), as shown in Figures 4b,c.

### Effects of RUNX2 overexpression on chondrocyte apoptosis and gene transcription profile

**FIGURE 5:**
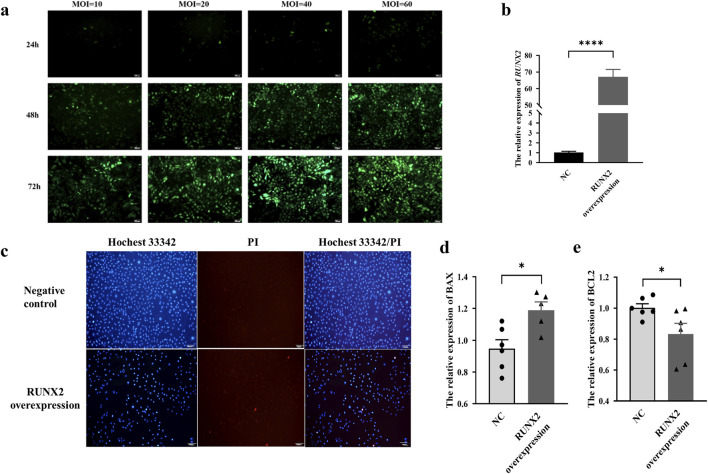
Effects of RUNX2 overexpression on chondrocyte apoptosis and necrosis. **(a)** The optimal multiple of infection (MOI) for RUNX2 overexpression. **(b)** Validation of RUNX2 overexpression efficiency by qRT-PCR. **(c)** Representative images of Hoechst 33342/PI staining in chondrocytes of RUNX2 overexpression. **(d,e)** The mRNA level of apoptosis-related genes (BCL-2, BAX) in the RUNX2 overexpression group. Data on the graph are shown as mean ± SD; * Compared with the control group, *P* < 0.05.

The MOI value was set to 40 for 72 h, and the RUNX2 overexpression stable cell line was screened and then verified by qRT-PCR (P < 0.0001), as shown in Figures 5a,b. The Hoechst 33342/PI staining showed that apoptotic and necrotic cells were increased in the *RUNX2* overexpression group (Figure 5c). Compared with the negative control group, the expression of *BAX* was increased, and *BCL-2* was decreased (*P* = 0.0127, *P* = 0.0449), which suggested that RUNX2 overexpression could cause chondrocyte apoptosis and then cause cartilage injury (Figures 5d,e).

**FIGURE 6:**
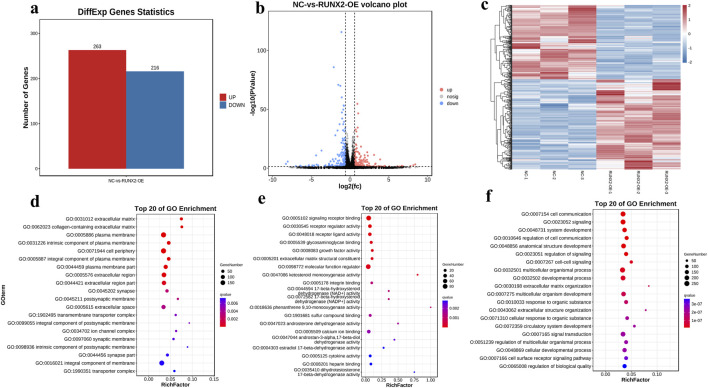
Differential and functional analysis of RUNX2 overexpression. **(a)** DEGs statistics in RUNX2 overexpression by RNA sequencing. **(b)** The volcano plot of DEGs between RUNX2 overexpression and the negative group. **(c)** The cluster heat map of DEGs between RUNX2 overexpression and the negative group. **(d–f)** The GO enrichment difference bubble plot of the top 20 DEGs [d cellular component (CC), e molecular function (MF), f biological process (BP)].

**FIGURE 7:**
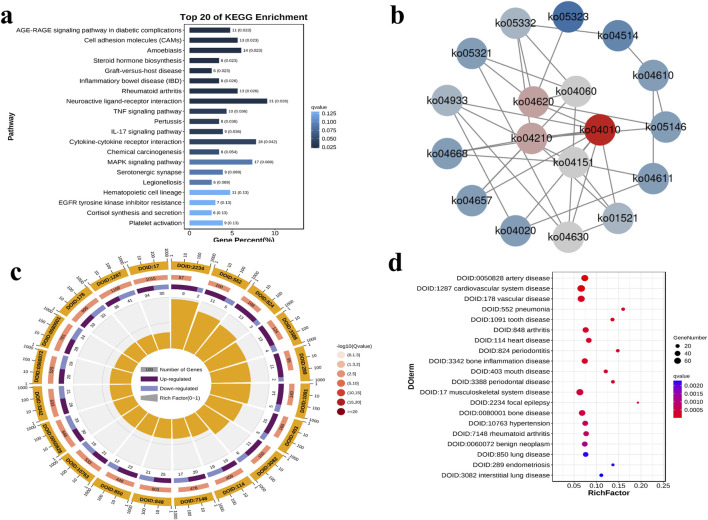
The KEGG enrichment pathway and disease ontology (DO) analysis of DEGs. **(a)** The KEGG enrichment bar diagram of the top 20 KEGG enrichment pathways. **(b)** The network diagram of the top 20 KEGG enrichment pathways. **(c)** The Do enrichment circle diagram of the top 20 DO enrichment. **(d)** The DO enrichment difference bubble plot of the top 20 DO enrichment.

Further, we analyzed the differentially expressed genes in the RUNX2 overexpression model by RNA-sequencing. The RNA-seq data passed all quality control criteria, ensuring sufficient sequencing depth and data reliability for downstream analyses. a total of 479 differentially expressed genes (DEGs) were obtained from the RUNX2 overexpression and negative control group, of which 263 genes were upregulated and 216 genes were downregulated (Figure 6a). The volcano plot and cluster heat map are shown in Figures 6b,c. DEGs functions were analyzed using GO analysis, which contains cellular component (CC), molecular function (MF), and biological process (BP). The GO analysis result showed that, in CC terms, the extracellular matrix, collagen-containing extracellular matrix, and plasma membrane were mainly enriched, as shown in Figures 6d–f. For MF, DEGs were primarily enriched in signaling receptor binding, receptor regulator activity, and receptor-ligand activity. For BP, DEGs were enriched mainly in cell communication, signaling, and system development. For KEGG enrichment pathway analysis in Figure 7a, the results showed that DEGs were primarily associated with the AGE-RAGE signaling pathway in diabetic complications, cell adhesion molecules (CAMs), amoebiasis, and were also involved in rheumatoid arthritis, TNF signaling pathway, and MAPK signaling pathway, among others. And there were many interactions between KEGG pathways (Figure 7b). Further, the relationship between the function of differential genes and diseases was analyzed by using the disease ontology (DO) database (Figures 7c,d). The DO result showed that DEGs were mainly included in various diseases, such as artery disease, cardiovascular system disease, pneumonia, arthritis, musculoskeletal system disease, bone disease, and rheumatoid arthritis etc.

## Discussion

KBD is a nutrition-related osteoarthropathy characterized by cartilage degeneration driven by chondrocyte death and ECM destruction. Although environmental risk factors have been linked to the etiology and pathogenesis of KBD, the molecular mechanisms underlying cartilage injury remain poorly understood. In the present study, H&E staining revealed acellular areas and chondrocyte necrosis in cartilage samples from KBD patients. Moreover, we observed reduced collagen mRNA expression, accompanied by increased ECM degradation and apoptosis in KBD cartilage. These findings indicate that chondrocyte necrosis, excessive apoptosis, and ECM degradation are hallmarks of KBD pathology, consistent with previous reports. (Zhang et al., 2018).

RUNX2 is essential for chondrocyte maturation and normal endochondral bone formation (Hallett et al., 2025). In this study, the proportion of RUNX2-positive cells in articular cartilage from KBD patients was higher than that in controls, with particularly strong expression in necrotic areas of the deep cartilage. *RUNX2* expression was also upregulated in the peripheral blood of KBD patients, suggesting that *RUNX2* may contribute to cartilage injury of KBD. Consistently, previous studies have shown that RUNX2 is upregulated in human osteoarthritic cartilage and in murine articular cartilage following joint injury (Catheline et al., 2019). Moreover, conditional knockout of RUNX2 in chondrocytes attenuated disease progression in an experimental OA mouse model (Liao et al., 2017). Together, these findings support a vital role for RUNX2 in KBD and highlight the importance of investigating factors that regulate RUNX2 expression.

DNA methylation can dynamically regulate gene expression in chondrocytes in response to environmental factors (den Hollander et al., 2015). Increasing evidence indicates that DNA methylation is closely associated with gene expression in diseases such as OA (Van Meurs, 2017; Bui et al., 2021), aging (Simpson and Chandra, 2021), and cancer (Xie et al., 2021; Conteduca et al., 2021). In this study, the methylation level of the *RUNX2* promoter in KBD patients was significantly lower than that in controls. Furthermore, inhibition of DNA methylation in chondrocytes using 5-aza-CdR demonstrated that reduced methylation was accompanied by increased *RUNX2* expression. Similarly, aberrant DNA methylation and elevated RUNX2 expression have been implicated in abnormal bone and cartilage development (Grcevic et al., 2010; Rice et al., 2018). Genome-wide association studies have demonstrated that elevated RUNX2 expression is associated with RUNX2 hypomethylation in OA (Rice et al., 2018; Song et al., 2018). Similarly, a negative correlation between RUNX2 methylation and gene expression has been reported in periodontal ligament osteoblasts (Ferreira et al., 2022).

Furthermore, previous studies have shown that the methylation and expression of *GPX3, DIO3* were altered in KBD patients and negatively correlated (Han et al., 2018; Li et al., 2020). These suggest that DNA methylation contributes to disease development by regulating gene expression and, more specifically, indicate that *RUNX2* hypomethylation leads to increased *RUNX2* expression in KBD. Excessive apoptosis has been recognized as a key pathological feature of KBD. In this study, we observed that *RUNX2* overexpression enhanced chondrocyte apoptosis, as evidenced by an increased apoptosis rate and upregulation of the pro-apoptotic marker *BAX*, alongside reduced expression of the anti-apoptotic marker *BCL-2*. These results suggest that elevated *RUNX2* expression promotes chondrocyte apoptosis in KBD. Consistent with this, recent studies have highlighted the central role of apoptosis in articular cartilage injury in KBD (Wu et al., 2024; Zhang et al., 2014). Furthermore, the DNMT3b/miR-29b/PTHLH/CDK4/RUNX2 axis has been implicated in OA-related chondrocyte apoptosis, further confirming the pathogenic role of RUNX2 in OA (Dou et al., 2020). Collectively, our findings support the conclusion that RUNX2 contributes to chondrocyte apoptosis in KBD.

KBD is thought to result from the combined influence of genetic predisposition and environmental factors, both of which have become important areas of research. Zhang et al. reported that KBD patients remain in a state of selenium deficiency, likely due to chronically low dietary selenium intake through the food chain (Zhang et al., 2023). Epidemiological studies have further shown that selenium levels in drinking water and wheat flour are significantly lower in KBD-endemic regions compared with non-endemic areas (Wu et al., 2024). However, it remains unclear whether such environmental risk factors contribute to KBD pathogenesis via RUNX2 upregulation. In this study, we found that selenium deficiency reduced *RUNX2* promoter methylation, leading to excessive chondrocyte apoptosis, whereas selenium supplementation reversed these effects. Recent evidence has demonstrated that low selenium levels can induce oxidative stress-mediated DNA damage, thereby impairing cell cycle regulation and membrane integrity and promoting apoptosis (Yara et al., 2015; Yang et al., 2020). Moreover, transcriptomic profiling has revealed that selenoproteins are dysregulated in KBD patients, with altered expression of *GPX3, DIO1*, and *TXNRD1* implicated in chondrocyte apoptosis through reduced antioxidant capacity (Yang et al., 2022a). Taken together, these findings suggest that selenium deficiency contributes to KBD pathogenesis by decreasing *RUNX2* methylation, thereby upregulating *RUNX2* expression and driving chondrocyte apoptosis.

In the *RUNX2* overexpression group, a total of 479 differentially expressed genes (DEGs) were identified, including 263 upregulated and 216 downregulated genes. Gene Ontology (GO) enrichment analysis revealed that these DEGs were predominantly associated with biological processes involving the extracellular matrix, collagen-containing extracellular matrix, and plasma membrane. KEGG pathway analysis further showed significant enrichment of DEGs in the TNF and MAPK signaling pathways, both of which are known to regulate cell proliferation, apoptosis, differentiation, and inflammation. For instance, TNF signaling activates caspase proteins, JNK, and the transcription factor NF-κB through receptor binding, thereby influencing cell growth, differentiation, apoptosis, and inflammatory responses (Fedorka et al., 2022; Zhao et al., 2021). Both clinical and experimental studies have shown elevated levels of inflammatory cytokines in KBD. Specifically, serum IL-1β and TNF-α levels are significantly higher in children with KBD compared with those from non-KBD areas, and similar increases have been observed in rats fed with a KBD-inducing diet (Tang et al., 2012). Furthermore, TNF-α stimulation induces the overexpression of TSG-6, which in turn significantly upregulates the expression of MMP1, MMP3, and MMP13, as well as aggrecan degradation, while markedly downregulating COL2A1 expression. These changes collectively promote extracellular matrix damage and degradation in chondrocytes from patients with KBD (Ning et al., 2022). Similarly, the MAPK signaling pathway modulates cell growth, proliferation, differentiation, and apoptosis via ERK, JNK, p38/MAPK, and ERK5 cascades, and its activation is critical for cartilage and joint development (Gunnell et al., 2010; Kim and Choi, 2015; Wang et al., 2017). Notably, abnormal expression of ERK and JNK has been observed in KBD patients, implicating MAPK dysregulation in KBD-related cartilage injury (Dai et al., 2017). Interleukin-1β (IL-1β) can activate the MAPK signaling pathway, thereby inducing the expression of catabolic enzymes that contribute to extracellular matrix (ECM) degradation in chondrocytes (Huang et al., 2019). Moreover, recent evidence indicates that dysfunction of the MAPK signaling pathway plays a critical role in T-2 toxin–induced chondrocyte injury, leading to impaired cell viability and accelerated ECM degradation (Yang et al., 2022b). DO analysis further indicated that these DEGs were enriched in categories such as arthritis, musculoskeletal system disease, bone disease, and rheumatoid arthritis. Collectively, these findings suggest that RUNX2 plays an indispensable role in the pathogenesis of KBD, likely through its involvement in key signaling pathways regulating cartilage homeostasis and chondrocyte survival.

In addition, We acknowledge that the relatively small number of patient samples used for cartilage analysis is a limitation of this study. This limited sample size may reduce the statistical power and generalizability of the findings. Nevertheless, the consistent trends observed among patients provide valuable preliminary insights into the pathological changes of cartilage in this condition. Future studies with larger cohorts are warranted to further validate and expand upon these findings.

## Conclusion

In summary, RUNX2 was upregulated in both cartilage and peripheral blood of KBD patients, with its increased expression associated with promoter hypomethylation. Micronutrient selenium deficiency contributed to RUNX2 overexpression by reducing methylation levels, thereby promoting cartilage injury, whereas Se supplementation reversed these effects. RUNX2 overexpression could led to excessive chondrocyte apoptosis, necrosis, and transcriptomic alterations, with differentially expressed genes predominantly enriched in the TNF and MAPK signaling pathways. Collectively, this study highlights the critical role of RUNX2 in regulating chondrocyte apoptosis and necrosis and provides new insights into the etiology and pathogenesis of KBD, suggesting RUNX2 as a potential target.

## Data Availability

The data presented in the study are deposited in the GEO repository, accession number GSE311894, available at: https://www.ncbi.nlm.nih.gov/geo/query/acc.cgi?acc=GSE311894.
