# Supplementary material for: Effects of selenium-mediated RUNX2 overexpression and its transcriptome alterations on Chondrocyte injury in Kashin Beck disease
Source: Front Cell Dev Biol. 2025 Dec 5;13:1687954. doi: 10.3389/fcell.2025.1687954 (PMC12714888; doi:10.3389/fcell.2025.1687954)
Supplement: Supplementary file 1 [file Table1.docx]

Table S1. Primers sequences used in qMSP-PCR

| Gene | Primers sequences (5′-3′) |
| --- | --- |
| *RUNX2* | MF: 5’-GTTTGAGGGCGGGTGGTAGTCGC-3’ |
|  | MR:5’-ACTACCCCGAAAAATCTAAATCG-3’ |
| *β-actin* | MF:5’-TGGTGATGGAGGAGGTTTAGAAGT-3’ |
|  | MR:5’-AACCAATAAAACCTACTCCTCCCTTAA-3’ |

Table S2. Primers sequences used in qRT-PCR

| Gene | Primers sequences (5′-3′) |
| --- | --- |
| *RUNX2* | Fw: TTCACCTTGACCATAACCGTC Rv: GGCGGTCAGAGAACAAACTAG |
| *COL2A1* | Fw: GGATGCCACACTCAAG Rv: TTGGGGTAGACGCAAG |
| *COL10A1* | Fw: GGGAGTGCCATCATCG Rv: AGGGTGGGGTAGAGTT |
| *ADAMTS4* | Fw: ATGGCTATGGGCACTGTCTC Rv: GTGTTTGGTCTGGCACATGG |
| *ADAMTS5* | Fw: CCTGCCCACCCAATGGTAAATC Rv: CGGCCTACATTCAGTGCCATC |
| *BAX* | Fw: CCTTTTGCTTCAGGGTTTCAT Rv: GAGACACTCGCTCAGCTTCTTG |
| *BCL2* | Fw: TGGGATGCCTTTGTGGAACT Rv: GAGACAGCCAGGAGAAATCAAAC |
| *β-ACTIN* | Fw: ATTGCCGACAGGATGCAGA Rv: GAGTACTTGCGCTCAGGAGGA |
| *DNMT1* | Fw: AGACTACGCGAGATTCGAGTC Rv: TTGGTGGCTGAGTAGTAGAGG |
| *DNMT3a* | Fw: CTGAAGGAGTATTTTGCGTGTG Rv: CTTCTGGGTGCTGATACTTCTC |
| *DNMT3b* | Fw: CCCATTCGAGTCCTGTCATTG Rv: TTGATATTCCCCTCGTGCTTC |

Table S3. Characteristics of the study population

|  | Characteristics | KBD | Controls | *P* |
| --- | --- | --- | --- | --- |
| Cartilage | Age*， mean±SD | 58.60±6.99 | 60.4±11.15 | 0.793 |
|  | Sex，female/male | 2/3 | 2/3 | / |
| Blood | Age， mean±SD | 58.20±7.59 | 57.50±7.33 | 0.907 |
|  | Sex，female/male | 8/12 | 9/11 | 0.835 |

/ indicates not applicable; no statistical test was performed due to small sample size.

* KBD (51,56,58,58,70 ); *Control (47,52,60,71,72 )

Table S4. The number of positive staining cells for RUNX2

| Group | Number of cartilage stratified cells (positive/total cells) | | | Total |
| --- | --- | --- | --- | --- |
|  | Superficial layer | Middle layer | Deep layer |  |
| Control | 9.56%  （196/2050） | 19.34%  (181/936) | 39.21%  (356/908) | 17.97%  （771/4291） |
| KBD | 50.81%  (814/1602) | 64.33%  (743/1155) | 80.03%  (593/741) | 60.07%  (355/2657) |
| χ^2^ | 764.807 | 424.354 | 278.295 | 1428.946 |
| *P* | ＜0.0001 | ＜0.0001 | ＜0.0001 | ＜0.0001 |
